# Methods and Measures to Assess Health Care Provider Behavior and Behavioral Determinants in Reproductive, Maternal, Newborn, and Child Health: A Rapid Review

**DOI:** 10.9745/GHSP-D-22-00407

**Published:** 2023-11-30

**Authors:** Leanne Dougherty, Sanyukta Mathur, Xaher Gul, Kathryn Spielman, Vandana Tripathi, Christina Wakefield, Martha Silva

**Affiliations:** a Population Council, Washington, DC, USA.; b Pathfinder International, Karachi, Pakistan.; c Population Council, Baltimore, MD, USA.; d EngenderHealth, New York, NY, USA.; e Manoff Group, Seattle, WA, USA.; f Tulane University, New Orleans, LA, USA.

## Abstract

**Background:**

Health care provider behavior is the outcome of a complex set of factors that are both internal and external to the provider. Social and behavior change (SBC) programs are increasingly engaging providers and introducing strategies to improve their service delivery. However, there is limited understanding of methods and measures applied to assess provider behavioral outcomes and strengthen provider behavior change programming.

**Methods:**

Using PubMed, we conducted a rapid review of published research on behaviors of health workers providing reproductive, maternal, newborn, and child health services in low- and middle-income countries (2010–2021). Information on study identifiers (e.g., type of provider), select domains from Green and Kreuter's PRECEDE-PROCEED framework (e.g., predisposing factors such as attitudes), study characteristics (e.g., study type and design), and evidence of theory-driven research were extracted from a final sample of articles (N=89) and summarized.

**Results:**

More than 80% of articles were descriptive/formative and examined knowledge, attitudes, and practice, mostly related to family planning. Among the few evaluation studies, training-focused interventions to increase provider knowledge or improve competency in providing a health service were dominant. Research driven by behavioral theory was observed in only 3 studies. Most articles (75%) focused on the quality of client-provider interaction, though topics and modes of measurement varied widely. Very few studies incorporated a validated scale to measure underlying constructs, such as attitudes and beliefs, and how these may be associated with provider behaviors.

**Conclusion:**

A need exists for (1) theory-driven approaches to designing and measuring provider behavior change interventions and (2) measurement that addresses important internal and structural factors related to a provider's behavior (beyond knowledge-enhancing training approaches). Additional investment in implementation research is also needed to better understand which SBC approaches are shifting provider behavior and improving client-provider interactions. Finally, theory-driven approaches could help develop empirically measurable and comparable outcomes.

## Abstract (precis)

There is a limited understanding of health care provider behavior change approaches and how they're being measured. This rapid review identifies methods and measures to understand opportunities and gaps in assessing and improving health care provider performance.

## Teaser

**Key Findings:**

- This rapid review identified opportunities and gaps in measurement of health care provider behavior by focusing on domains that correspond to factors that influence provider behavior and service provision and incorporate elements beyond provider competency and skills.
- Of the studies included in this review, 65% focused on providers' ability (knowledge, skills, and access to clinical training); 70% focused on predisposing factors (attitudes, beliefs, and perceptions); 57% focused on enabling factors (resources and skills required to make behavioral changes); and 36% focused on reinforcing factors (e.g., peer support and supervisor feedback).
- Most studies did not test associations between behavioral determinants and provider behaviors.

**Key Implications:**

- Program implementers, donors, and researchers should support the evaluation of provider behavior change programs to shift the evidence base from one that is focused on describing behaviors to one that uses theory-driven approaches to understand behavioral antecedents and impacts of behavior change interventions on provider behaviors and their drivers.
- Evaluators should develop and apply validated measures of provider behavior to enable comparable learning and support policymakers to target quality improvement and invest in evidence-based behavior change programs.

## INTRODUCTION

Broadly defined, health care providers are individuals who provide services, products, or information with the aim of promoting, protecting, and improving health.^1^ The 2013 Recife Political Declaration on Human Resources for Health established an ambitious agenda for health workforce development so that “all people everywhere have access to a skilled, motivated health worker, within a robust health system.”^2^ The Global Strategy for Human Resources for Health that followed this declaration outlined multiple objectives related to optimizing health care worker performance, aligning investments in human resources, building capacity at multiple levels, and strengthening data on human resources for health for monitoring and ensuring accountability.^3^ Within this context, there emerged a growing demand for information on the health workforce, including achieving consensus on a core set of indicators and data for monitoring the availability, distribution, and training of providers.^4^ However, a recent systematic review found capacity-strengthening for primary health care was predominantly conceptualized in relation to knowledge and clinical skills^5^ with limited reflection on how this translates into competence, which is the combination of skills, knowledge, interpersonal and intrapersonal factors, and behavior that providers exercise in delivering high-quality care.^6^ Further, additional systematic reviews have highlighted factors beyond knowledge and skills that influence provider motivation, including financial incentives, career development, and adequate resources and their links to health worker retention and quality care.^7^^,^^8^ Indeed, health care provider behavior—which includes a range of actions, from facility management and adherence to clinical protocols to supervision and client-provider interaction—is the outcome of a complex set of factors that are both internal (e.g., attitudes, values, and beliefs) and external (e.g., supervisor support, access to professional development, and supportive workplace environment) to the provider.^9^

In response, health and development programs are increasingly leveraging social and behavior change (SBC) approaches to better engage providers and introducing strategies and tools, such as the “Provider Behavior Ecosystem Map,” that reflect on the entire ecosystem of influencers and ensure that they are considered in intervention strategies.^10^ Better engaging providers goes beyond increasing knowledge and skills and includes addressing provider behaviors and their underlying determinants. However, there is limited understanding of how to identify and measure critical drivers of provider behaviors, which, in turn, makes it challenging not only to develop health care provider behavior change (PBC) interventions that are guided by empirical evidence but also to measure their impact. The recent consensus-driven global Research and Learning Agenda for Advancing PBC Programming identified the need for more evidence to inform PBC strategies, including the need for (1) comparable and comprehensive measurement of the quality of client-provider interactions from client and provider perspectives; (2) measurement of provider attitudes, perceptions of norms, and biases that influence their performance and adherence to timely and respectful client-centered care practices; and (3) measurement of the social and structural environment within which providers operate.^11^

There is limited understanding of how to identify and measure critical drivers of provider behaviors, making it challenging to develop PBC interventions guided by empirical evidence and measure their impact.

Globally, countries face challenges in varying degrees related to the performance of their health workforce. Addressing these challenges requires a process where the problem and its determinants are clearly defined in measurable terms. The Breakthrough RESEARCH project—funded by the U.S. Agency for International Development—and the SBC for Service Delivery Working Group, a community of professionals committed to improving the practice of integrating SBC across the service continuum,^12^ collaborated to address these evidence gaps by conducting a rapid review of the published peer-reviewed literature to determine what methods and measures have been used to assess provider behavioral outcomes in the area of reproductive, maternal, newborn, and child health (RMNCH). This rapid review aims to identify and describe methods and measures related to provider behaviors and their drivers and to identify gaps in measurement and opportunities that can inform future PBC strategies.

## METHODS

### Information Sources and Search Strategy

In line with Cochrane guidance, we conducted a rapid review of the peer-reviewed literature from January 2000 through May 2021 by searching the PubMed database.^13^ We used a wide range of terms, including National Library of Medicine Medical Subject Headings related to 5 categories: (1) low- and middle-income countries; (2) RMNCH; (3) health workers, providers, and community health workers, including accredited social health activists; (4) health worker (provider) behaviors; and (5) (health) provider behavior interventions. We included English, French, Spanish, and Portuguese language articles because members of the review team were able to critically review articles in these languages. A full list of search terms applied is available in Supplement 1. We excluded reviews, commentaries/editorials, and news articles.

### Study Selection

**FIGURE 1:**
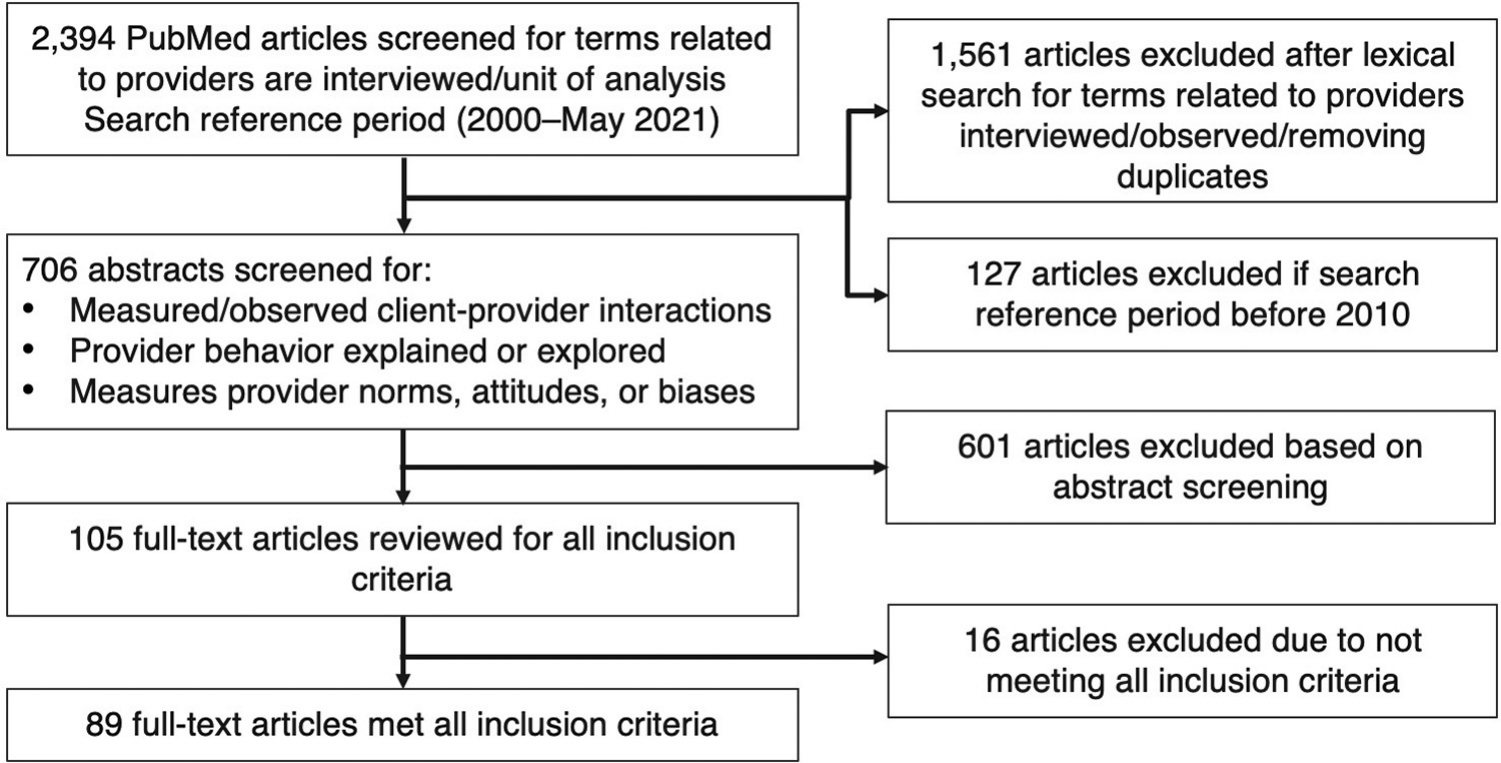
Schematic of Search Strategy and Results on Assessing Health Provider Behaviors

We imported citations into Zotero software and removed duplicates. The initial screen was done in collaboration with a second team engaged with the SBC for Service Delivery Working Group (H. Hancock and O. Carlson, unpublished data, May 2021). Reviewers screened titles of the articles retrieved (N=2,394) to exclude articles that did not take place in low- and middle-income countries, did not focus on RMNCH, or did not interview or observe providers (Figure 1). Following the initial title screen, the study team refined the study reference period to include articles from January 2010 through May 2021 to focus on more recent PBC measurement efforts, resulting in 706 articles that advanced to abstract screening.

During the abstract screening, a single reviewer assessed articles to determine if (1) client-provider interactions were measured/observed, (2) provider behavior was explained or explored from the provider perspective, or (3) provider norms, attitudes, or biases were measured. A sample of 10% of articles was double screened by 2 study team members to ensure agreement with the inclusion criteria. Any decisions that did not meet consensus were discussed and resolved. The study team then completed full-text extraction of the 105 abstracts that met the inclusion criteria, during which 16 articles were further excluded for failing to meet the inclusion criteria, resulting in 89 articles for full review and data extraction.

### Data Extraction

We designed and piloted a data extraction table using Google Sheets to record study identification (title, author, and year), health area, geographic information (country and World Health Organization [WHO] region), type of health worker, what types of provider behavior/client-provider interactions were measured, and whether the behavior was self-reported or observed (Supplement 2). We also categorized whether the articles measured provider ability and select domains using a well-established behavior change model, PRECEDE-PROCEED, as an organizing framework for the analysis.^14^ We used this behavior change model as it considers individual-level determinants and their interactions with system-level determinants. These selected domains correspond to known factors influencing provider behavior and illustrate elements beyond health worker ability (i.e., competency and skills) that influence service provision or, specifically, client-provider interactions.^15^ We did not analyze associations between select domains and behaviors.

For each article, we captured (1) predisposing factors (e.g., individual attitudes, beliefs, and perceptions); (2) reinforcing factors (e.g., those that follow a behavior and determine whether, for example, a health worker receives positive [or negative] feedback from their supervisors); and (3) enabling factors, which are the resources and skills required to make desired behavioral and environmental changes (e.g., availability of medical supplies enables a health worker to offer health services).^14^ The categorization framework was developed a priori with the understanding that it would be expanded if additional information emerged during content extraction (Table 1). For each measurement category, we assessed how the item was measured (e.g., single item, qualitative, or scale) and extracted text on specific measures applied. We extracted information on the type of study, study design, whether the study included an intervention, and whether the design or measurement was driven by an empirical theory. All study authors contributed to data extraction, which involved an iterative process to achieve consensus on extraction criteria.

**TABLE 1.** Illustrative Examples of Behaviors and Behavioral Determinants by Domain Used for Data Extraction^16^

Domain	Examples
Provider behavior/client-provider interaction	Client reception and admission Clinical management (e.g., diagnostics, care/treatment, and referral) Person-centered care (e.g., respectful care, ensuring privacy, and confidentiality) Recordkeeping and stock management
Ability	Assesses provider knowledge and awareness Assesses provider skills Exposure to training
Predisposing	Attitudes toward certain products, services, or workplace Provider attitudes and biases toward clients Perceptions of control, self-efficacy, and agency for delivering services
Reinforcing	Supervision, including supervision frequency, feedback, and appreciation Financial incentives Professional growth opportunities Peer support and facility-level norms
Enabling	Quality of physical infrastructure (e.g., water and electrical source and overall cleanliness) Management of staff (e.g., number of staff and management meetings) Commodities and services (e.g., availability of contraceptives and examination room equipment) Space (e.g., whether exams occurred in a separate room or behind a curtain) Counseling materials (e.g., number of counseling aids and counseling protocols available) Health information systems and client records

### Data Synthesis and Analysis

We conducted descriptive analyses of closed-ended questions in the extraction sheet using Stata 16. Open-ended responses in the extraction table were grouped and synthesized by measurement domains.

## RESULTS

### Study Characteristics

Table 2 provides a description of study characteristics. Articles primarily focused on family planning (FP) and reproductive health (60%). Among articles that addressed multiple health areas, the majority addressed FP and maternal and child health or FP and HIV/AIDS. About a quarter of the articles reviewed focused on maternal health, specifically prenatal and delivery care.^17^^–^^20^ Very few articles focused on child health, including newborn care, managing febrile cases, and referral for diarrhea and acute respiratory illness.^19^^–^^22^ More than half of the articles reviewed were from the Africa region (60%), with many focusing on South Africa, Nigeria, Uganda, and Tanzania. The Southeast Asia region contributed 16% of articles to the analysis, primarily from India. There was limited representation from non-Anglophone countries, despite including French, Spanish, and Portuguese language articles in the search criteria. We applied the WHO definition of health worker types to categorize the study subjects.^1^ A third of the studies focused exclusively on health professionals (e.g., medical doctors, nurses, midwives, and pharmacists), and another 40 studies (45%) addressed multiple types of providers. In studies addressing multiple types of providers, health associate professionals (n=25) and health management and support personnel (n=18) were also mentioned often.

**TABLE 2.** Characteristics of Studies on Health Care Provider Behaviors in Reproductive, Maternal, Newborn, and Child Health, 2010–2021

	No. (%) (N=89)
Health area	
Child health	1 (1.1)
Family planning/reproductive health	53 (59.6)
Maternal health	17 (19.1)
Multiple areas	18 (20.2)
World Health Organization region	
Africa	53 (59.6)
Americas	5 (5.6)
Eastern Mediterranean	5 (5.6)
European	4 (4.5)
South East Asia	14 (15.7)
Western Pacific	4 (4.5)
Multiple	4 (4.5)
Type of provider^1^	
Health professionals	29 (32.6)
Health associate professionals	10 (11.2)
Multiple	40 (44.9)
Not specified/other	10 (11.2)

### What Study Methods Were Used?

Table 3 presents the research methods used in articles assessing provider behaviors. The highest percentage (42%) of studies were cross-sectional in design and approximately half were qualitative or mixed methods. Among the mixed methods studies, the majority supplemented quantitative cross-sectional surveys with qualitative in-depth interviews or used service provision assessment methods, which include facility assessments, client-provider observations, and client exit interviews. A few studies also incorporated mystery clients^23^^–^^25^ and videotaped observations.^26^ Two studies used the Demographic and Health Survey Service Provision Assessments datasets to conduct multicountry analyses.^21^^,^^27^ Two studies focused on developing measures: 1 study developed an integration index indicator to assess integration of FP and immunization services,^28^ and a second study developed measurement tools to assess how women are treated during facility delivery.^17^ Very few studies were evaluations of provider-focused behavioral interventions (less than 20%), and the interventions described were predominantly training-focused. Of the 12 evaluation-focused articles, only 2 studies included a comparison group,^18^^,^^29^ while 4 evaluations were pre-post assessments without a control.^23^^,^^27^^,^^30^^,^^31^ The remaining evaluation study designs were intervention only, mixed methods,^24^^,^^32^^–^^35^ and qualitative.^36^ The mixed methods evaluations included a range of methodological approaches, such as mystery clients, pre-post assessments, services statistics, in-depth interviews, and observations. Only 1 of the quantitative evaluations described how the sample design was powered to measure differences over time and study group.^23^

**TABLE 3.** Methods Used in Studies on Health Care Provider Behaviors in Reproductive, Maternal, Newborn, and Child Health, 2010–2021

	No. (%) (N=89)
Study design	
Cross-sectional	37 (41.6)
Mixed methods	20 (22.5)
Qualitative	24 (27.0)
Pre-post no control	4 (4.5)
Other quantitative design	4 (4.5)
Study type	
Descriptive/formative	76 (84.4)
Evaluation	12 (13.3)
Measurement development	2 (2.2)
Intervention	
None	74 (82.2)
Training	8 (8.9)
Multiple	5 (5.6)
Other	3 (3.3)

**FIGURE 2:**
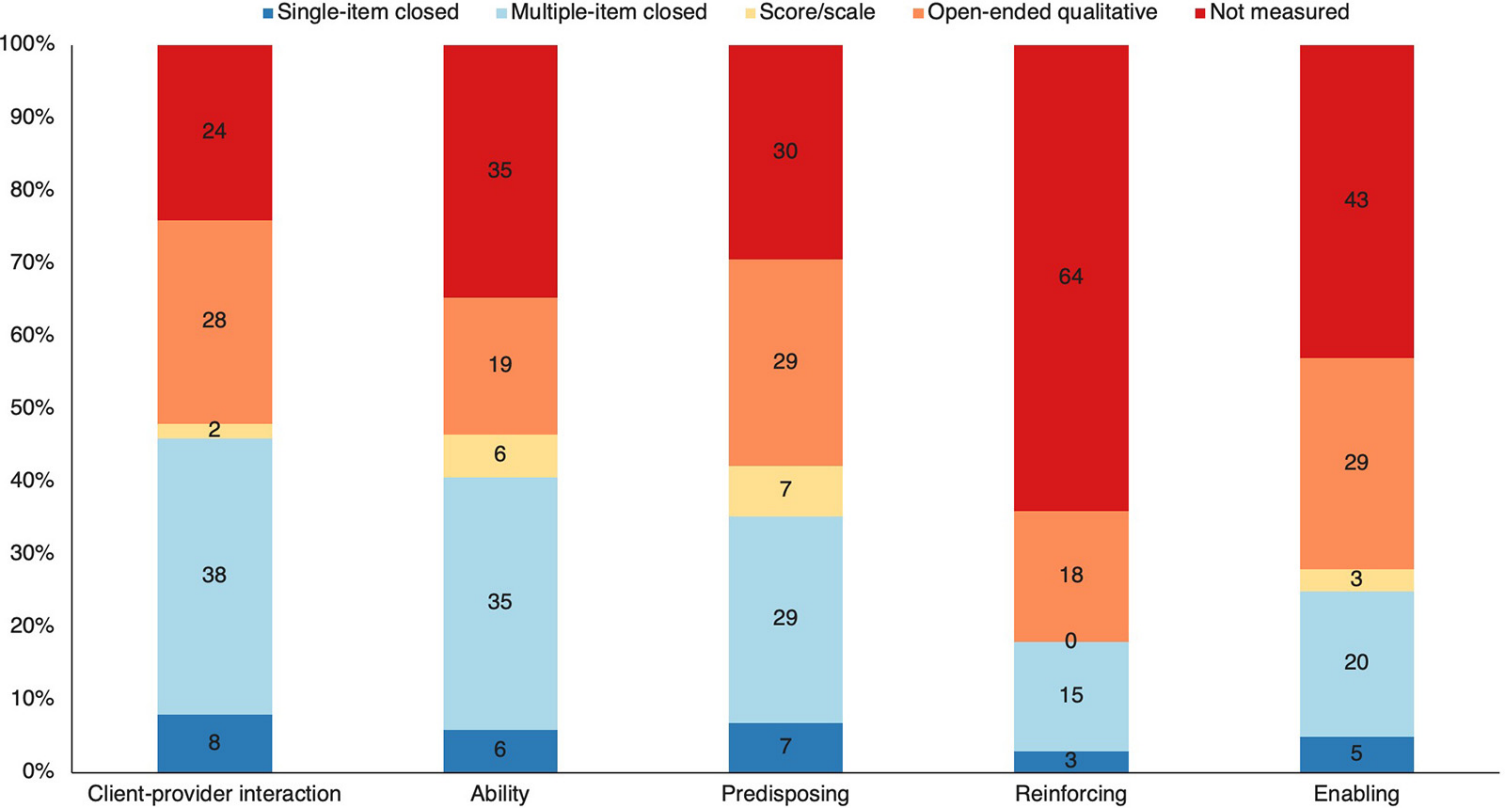
Proportion of Articles That Measured Provider Behavioral Determinants by Type of Measure (N=89)

Across all domains, the majority of studies used multiple-item closed-ended questions or qualitative measures (Figure 2). Few articles across all domains used and/or reported on the internal reliability of scales.^29^^,^^37^^–^^40^ Among the 89 studies reviewed, 76% assessed client-provider interactions, 65% assessed ability, 70% assessed predisposing factors, 36% assessed reinforcing factors, and 57% assessed enabling factors (Figure 2).

### What Types of Client-Provider Interactions Were Measured?

Among the 89 studies reviewed, 76% assessed client-provider interactions. Client-provider interaction was measured primarily through multiple-item closed-ended questions (38%) or qualitative methods (28%), and a small number of studies used a single-item question (8%) or applied a composite measure, such as an index or scale (2%). Several studies focused on the quality of client-provider interactions, including assessments of respect toward clients and their perspectives/opinions/concerns, verbal and physical abuse of clients, emotional support and empathy toward clients, confidentiality and privacy during interactions, locus of decision-making and consent for care, and communication style with clients. Commonly measured themes included documentation of clinical care practices during client visits and counseling about a particular service or product. Among articles focused on FP, nearly half measured provider behaviors related to contraception counseling and provision of contraceptives,^27^^,^^29^^,^^33^ with several of these focused specifically on emergency contraception.^40^^–^^42^ A few articles focused on counseling and provision of safe abortion care.^30^^,^^43^^–^^46^ Several articles also reflected on contraceptive counseling for a subgroup of clients, specifically youth.^25^^,^^47^^,^^48^ Among articles focused on maternal health, a number focused specifically on the provision of respectful treatment during delivery.^49^^,^^50^ Of the limited articles focused on child health, 1 focused on adherence to best practices for newborn care^19^ and another focused on good medical practice for sick children.^21^

### How Were the Domains Influencing Provider Behavior Measured?

#### Ability

Approximately two-thirds of the studies (n=58) assessed provider ability, primarily measured through multiple-item closed measures (35%) and qualitative measures (19%), while only approximately 6% of studies used single-item closed measures or scores/scales (Figure 2). Studies measuring ability included the knowledge or skills to provide RMNCH services and access to clinical training to reinforce these elements. Several of these studies applied knowledge, attitude, and practice surveys. Commonly reported measures of ability consist of knowledge and awareness, with only a handful of studies reporting on clinical skills and application or nonclinical behavior (e.g., respectful care and quality of interaction with clients). Measurement of clinical care practices, often collected through direct observations, assessed the accuracy or completeness of clinical care provided for a particular client (e.g., postpartum hemorrhage care). Among studies focused on provision of reproductive health services, providers reported (or were observed) to assess their knowledge to provide counseling and administer a range of contraceptive methods.^33^^,^^40^^,^^47^ Several studies examined knowledge and ability to counsel and administer specific methods (e.g., emergency contraception pills,^41^^,^^42^^,^^51^ intrauterine devices,^52^^–^^55^ and vasectomy^24^^,^^56^). Several studies focused on knowledge and skills related to performing abortions.^30^^,^^37^^,^^45^^,^^57^ Fewer studies assessed knowledge and skills to provide maternal and child health services. Two studies focused on child health, including knowledge of diarrhea and dehydration symptoms and appropriate recommendation of oral rehydration and antibiotics.^23^^,^^58^ A few studies addressed maternal health services, including knowledge of common obstetric ailments, recommended treatment, and appropriate referral.^18^^,^^59^^,^^60^

Approximately two-thirds of the studies assessed provider ability, primarily measured through multiple-item closed measures and qualitative measures.

#### Predisposing Factors

Of the 89 articles reviewed, 70% (n=62) measured predisposing factors. These measures were primarily captured through self-report, although 25 studies also or exclusively assessed or inferred attitudes and perceptions through observation, including by mystery clients. As shown in Figure 2, nearly equal numbers of studies measured predisposing factors through multiple-item closed-ended questions and qualitative or open-ended questions (approximately 30% each). Predisposing factors were rarely measured through single-item questions (3%). Scales were used in 7% of included studies; for example, the Attitude Toward Disabled Persons scale was used to measure attitudes of health care providers toward people with disabilities,^61^ and the Stigmatizing Attitudes, Behaviors and Actions Scale was applied to measure attitudes toward abortion.^62^ The majority captured provider attitudes, beliefs, and perceptions. Provider attitudes measured were varied and included attitudes toward a specific service or product and attitudes toward clients. Attitudes toward work, including job satisfaction, are summarized under the reinforcing domain. Among articles measuring attitudes toward a specific service or product, several focused on a specific contraceptive method (e.g., vasectomy,^24^^,^^56^ emergency contraception pills,^41^^–^^43^^,^^51^^,^^63^^,^^64^ and intrauterine devices^53^^,^^55^) or a specific service, such as abortion.^30^^,^^44^^,^^57^^,^^62^ While few articles measured attitudes consistently across countries and health areas, an exception was regarding medical eligibility criteria for contraception. Several studies included measures to assess a provider's potential biases in providing contraception to women who were unmarried, nulliparous, young, or did not provide their partner's consent.^47^^,^^52^^,^^54^^,^^55^^,^^65^^,^^66^ Among the limited maternal health articles, provider attitudes toward clients focused on sociodemographic factors, such as education and wealth (e.g., “It is easier assisting educated women when they come for maternal and neonatal care than women who are not educated” and “Some providers at this facility treat women of low social status more poorly than other women of higher status”).^67^ Other predisposing factors, such as personal experience, motivation, confidence, and self-efficacy and intention to act, were measured much less frequently.

#### Reinforcing Factors

About one-third of the articles (n=32) measured the reinforcing domain, of which half (n=16) used qualitative measures. Only 1 study examined reinforcing factors using a structured index (provider support environment index and management index).^27^ Most measures focused on some form of peer support and workplace norms. Among those articles focused on peer support, 2 incorporated multiple-item closed-ended Likert scale questions, such as, “People I know and respect think I should talk to HIV patients about their desires to have children.”^38^^,^^39^ Single-item close-ended questions, such as, “I feel most of my colleagues are respectful of patients when providing maternal and neonatal health care,” were used to assess perceived workplace norms.^67^ Several articles reviewed measured the frequency and quality of supervision and mentorship.^29^^,^^35^^,^^69^ A few articles measured attitudes toward work or workload and included Likert-type questions that assessed agreement with remuneration, equipment availability, workload, harmony in the workplace, and management.^18^^,^^37^^,^^67^ Others considered the existence of incentives, including appreciation, reward, or other monetary incentives.^31^^,^^60^^,^^69^ Two articles touched on job satisfaction, including emotional satisfaction with the job.^36^^,^^37^ A multicountry study leveraging the Demographic and Health Survey Service Provision Assessment data generated 2 indicators to measure elements in the reinforcing domain.^27^ First, the study created a provider-supportive environment binary indicator that assigned a value of 1 if any of 3 elements were present: clear job description, knowledge of opportunities for promotion, or availability of performance incentives. A management index indicator was also created to assess facility management practices fulfilled in each facility, including regular quality assurance reviews and supervisory visits.

#### Enabling Factors

A little more than half (57%) of the articles reviewed measured enabling factors. Half of these studies used qualitative (30%) or multiple-item closed-ended (20%) measures to assess enabling factors (Figure 2). Fewer than 5% used single-item closed-ended measures or scales. Among studies that applied quantitative measures, only a few took a comprehensive approach to assessing the enabling domain. One study developed 2 indexes to assess structural factors in the enabling domain: (1) an infrastructure index to measure the proportion of 20 supply-side factors present in each facility, including the availability of a functional ambulance and uninterrupted essential drug supply over the past month, and (2) an equipment index composed of 7 items to measure the proportion of equipment essential for visits for antenatal care.^27^ A study on provider perspectives of postabortion care in Tanzania measured service availability, human resource capacity, service delivery environment, availability of supplies and contraceptives, infection prevention and waste management, and availability and completeness of the health information system.^46^ One study incorporated structural measures, including availability of FP commodities and drugs; general clinic supplies; reagents; infrastructure including privacy; availability of information, education, and communication and visual aids; clinical protocols/policies; clinical information systems; and the number of facility staff available.^29^ Availability of resources, both commodities and staff, were elements of the enabling domain that were more routinely measured.^47^^,^^49^^,^^52^^,^^54^^,^^69^

#### Measuring Multiple Domains

The use of the PRECEDE-PROCEED model as an organizing framework provided an opportunity to highlight instances where studies addressed multiple domains influencing provider behavior. In total, 9 studies incorporated all PRECEDE-PROCEED domains and measured provider behavior and ability.^32^^,^^38^^,^^62^^,^^67^^,^^70^^–^^74^ Among these 9 studies, 3 used qualitative methods, 3 used mixed methods, and 3 used quantitative cross-sectional methods. Only 1 study explicitly applied behavioral theory.^70^ Several studies relied on qualitative research embedded within a larger research agenda to develop questions^38^ or relied on a review of the literature^62^^,^^67^^,^^73^ to develop study instruments.

### Is the Measurement Theory Driven?

Less than 10% (n=8) of articles were guided by a conceptual framework or were theory driven. Given that assessment of provider behavior has traditionally been viewed through the lens of health systems strengthening and quality-of-care frameworks, we did identify a couple of articles that acknowledged WHO's guidelines for essential elements of clinical care and Rowe's framework for explaining health worker practices,^27^ as well as Donabedian's quality-of-care framework.^29^ We also note the application of the theory of clinical reasoning, the Dreyfus model of skill acquisition,^52^ and participatory systems analysis for group model building in some studies.^75^ However, these frameworks do not reflect on provider behavioral determinants, such as attitudes, self-efficacy, and perceived norms. Among those studies that explicitly incorporate empirically driven behavioral theory, we found an ecological adaptation of the information, motivation, and behavioral skills model^39^; the social cognitive theory^70^; and the theory of planned behavior.^61^ The social ecological theory was also identified as an organizing framework.^46^

### Are the Studies Linking Determinants to Provider Behavior?

Approximately half (56%) of the studies reviewed assessed client-provider interaction using quantitative methods. We assessed whether these studies measured associations between behavioral determinants and provider behavior. Overall, most studies were cross-sectional and descriptive and did not test associations between behavioral determinants and provider behaviors.^19^^,^^23^^,^^32^^,^^40^^–^^44^^,^^53^^,^^54^^,^^64^^,^^66^^,^^67^^,^^69^^,^^74^^,^^76^^–^^78^ Several mixed methods studies used quantitative measures to assess provider behavior and incorporated qualitative methods to describe behavioral determinants influencing provider behaviors.^46^^,^^59^ Among the few studies where associations between behavioral determinants and provider behavior were assessed, the majority considered predisposing factors such as knowledge and attitudes related to specific methods (e.g., emergency contraception and no-scalpel vasectomy),^24^^,^^51^^,^^79^ services (e.g., postabortion care and referral for high-risk clients),^60^^,^^73^ or clients' attributes^61^ and their association with service provision. Only 2 studies considered reinforcing and enabling factors and their association with provider behaviors, specifically quality of care and provider performance.^27^^,^^68^

## DISCUSSION

This rapid review establishes a foundation of evidence on measures related to provider behavior for RMNCH services and identifies opportunities and gaps in measurement that can inform future PBC strategies. The review identifies several promising studies that have aimed to understand the complex environment where providers operate by capturing information across multiple domains. These studies have endeavored to measure the predisposing, reinforcing, and enabling domains, as well as providers' ability and behavior.^32^^,^^39^^,^^62^^,^^67^^,^^70^^–^^74^ Additionally, several studies have contributed measurement tools that can be used to frame and strengthen our understanding of (1) treatment of women during facility-based childbirth,^16^ (2) validated quality-of-care scales related to FP and reproductive health,^19^^,^^27^^,^^38^ and (3) health care provider job satisfaction.^37^ Finally, while there were few standardized measures to draw from, a number of studies developed multiple-item closed-ended questions through in-depth literature reviews and formative research to ensure contextual factors were reflected in the research.

The review identifies several promising studies that have aimed to understand the complex environment where providers operate by capturing information across multiple domains.

Overall, most studies were descriptive or formative in nature and were largely composed of qualitative and cross-sectional studies. While these studies provide valuable information to support programs by describing relevant features of the clinical context, including current care practices and provider attitudes, they are limited in their ability to assess change over time and cohorts of providers. The limited methodological approaches may reflect challenges in establishing a facility sampling strategy, linking client-provider observations to facility-level determinants, as well as recognizing the need to capture the domains through different methodologies such as observations, client-exit interviews, facility assessments, and provider interviews, which are not always feasible given some facilities have low client volume or providers are busy and unavailable to participate in interviews.^80^ We also found limited application of methodologies, such as mystery clients that can provide an objective measure of provider performance and provider surveys that can contribute to an understanding of the individual behavioral determinants influencing behaviors.^81^^,^^82^

There were few standardized measures that emerged during the review that could be applied for learning across settings. Despite the fact that attitudes are multidimensional and should be grounded in qualitative formative research and considered across multiple domains, the majority of studies assessed provider behavioral determinants through multiple-item closed-ended questions, and only a few based these questions on literature, qualitative formative research, or empirically grounded scales. Validated scales were rarely used to measure latent constructs critical to informing PBC interventions, such as provider bias.^83^ Despite the existence of such validated scales in the areas of FP and sexual and reproductive health, we found minimal application to providers in the studies reviewed.^84^^–^^87^

Finally, while many studies incorporated multiple domains and constructs related to provider performance, the majority did not refer to an explicit theoretical foundation and, in particular, behavioral theory. Some of the articles that assessed provider performance were grounded in Donabedian's quality-of-care framework^88^ and WHO's health system framework.^89^ However, these frameworks fail to acknowledge how individual behavioral determinants interact with systems-level determinants and can influence provider performance. Without an explicit theoretical foundation, research with and measurement of providers is more likely to miss the empirically grounded determinants of provider behavior and comprehensively assess these determinants. For instance, use of a behavioral theory, such as information, motivation, and behavioral skills model for PBC research, would yield evidence on several key dimensions to inform and evaluate individual-level behavior change, including on provider attitudes, knowledge and skills, awareness, intention, motivation, and outcome expectation. Similarly, incorporating the theory of planned behavior that acknowledges perceived behavioral control may identify instances where a provider may not feel they can practice a behavior because of barriers presented by the health system. As a result, even among the limited evaluations reviewed, most interventions operated under the assumption of a narrow supply-side framing and considered training sufficient to increase knowledge and skills rather than applying a behavior change lens to providers as individuals. In the future, to create a more comprehensive picture of the determinants influencing provider behavior, program implementers and researchers should consider applying both a framework to capture the system-level determinants and a behavioral theory to capture individual determinants.

Without an explicit theoretical foundation, research with and measurement of providers is more likely to miss the empirically grounded determinants of provider behavior and comprehensively assess these determinants.

### Limitations

There are several limitations that should be considered in the context of this rapid review, which uses a less comprehensive approach than a systematic review. We did not include studies from the gray literature or from high-income countries. The review also did not identify many studies on provider behavior related to child health, which may be a function of the search terms. Given the limited time for the review, the review team did not review references in articles that met the inclusion criteria. As a result, salient findings outside our search criteria are not reflected in these findings. Because the objective was to understand the breadth of methods and measures applied and given the diversity of articles captured during this review, we did not assess or weight the quality of methods and measures applied nor the associations between determinants and behaviors and are also unable to draw conclusions on ideal measures for assessing PBC interventions. Further, we were also not seeking to categorize the specific underlying factors critical to a particular provider behavior. Rather, we focused on how those factors and corresponding outcomes were measured, if at all. Subsequent research may need to focus specifically on developing validated, reliable measures for evaluating PBC interventions across health areas. Finally, in this review, we focused solely on research done with providers; future reviews should focus on the client perspectives, as well as how larger community norms affect client-provider interactions, including fear of retribution and social sanctions, to have a better understanding of research on provider behaviors.

## CONCLUSION

This review supports a call to action for programmers and researchers to advance evidence generation through theory-driven, systematic measurement of PBC programs. The design of interventions and research should incorporate a behavioral theory approach that recognizes and addresses the importance of internal and structural factors related to a provider's behavior and identifies empirically measurable outcomes that are comparable across programs and contexts. Measurements of core concepts of provider behavior are necessary to concretely assess and address provider performance.

## Supplementary Material

GHSP-D-22-00407-supplement-2.xls

GHSP-D-22-00407-supplement-1.pdf

## Notes

**Peer Reviewed**

**First published online:** July 24, 2023.
