# Supplementary material for: Methods and Measures to Assess Health Care Provider Behavior and Behavioral Determinants in Reproductive, Maternal, Newborn, and Child Health: A Rapid Review
Source: Glob Health Sci Pract. 2023 Nov 30;11(Suppl 1):e2200407. doi: 10.9745/GHSP-D-22-00407 (PMC10698233; doi:10.9745/GHSP-D-22-00407)
Supplement: GHSP-D-22-00407-supplement-1.pdf [file GHSP-D-22-00407-supplement-1.pdf]

Supplement to: Dougherty L, Mathur S, Gul X, et al. Methods and measures to assess health provider behavior and behavioral determinants in reproductive, maternal, and child health: a rapid review. *Glob Health Sci Pract*. 2023;11(Suppl 3):e2200407. https://doi.org/10.9745/GHSP-D-22-00407

Supplement 1. Search Terms Used

Category 1: Geography

("afghanistan"[Text Word] OR "albania"[Text Word] OR "algeria"[Text Word] OR "american samoa"[Text Word] OR "angola"[Text Word] OR "antigua"[Text Word] OR "barbuda"[Text Word] OR "argentina"[Text Word] OR "armenia"[Text Word] OR "armenian"[Text Word] OR "aruba"[Text Word] OR "azerbaijan"[Text Word] OR "bahrain"[Text Word] OR "bangladesh"[Text Word] OR "barbados"[Text Word] OR "belarus"[Text Word] OR "byelarus"[Text Word] OR "belorussia"[Text Word] OR "byelorussian"[Text Word] OR "belize"[Text Word] OR "british honduras"[Text Word] OR "benin"[Text Word] OR "dahomey"[Text Word] OR "bhutan"[Text Word] OR "bolivia"[Text Word] OR "bosnia"[Text Word] OR "herzegovina"[Text Word] OR "botswana"[Text Word] OR "bechuanaland"[Text Word] OR "brazil"[Text Word] OR "brasil"[Text Word] OR "bulgaria"[Text Word] OR "burkina faso"[Text Word] OR "burkina fasso"[Text Word] OR "upper volta"[Text Word] OR "burundi"[Text Word] OR "urundi"[Text Word] OR "cabo verde"[Text Word] OR "cape verde"[Text Word] OR "cambodia"[Text Word] OR "kampuchea"[Text Word] OR "khmer republic"[Text Word] OR "cameroon"[Text Word] OR "cameron"[Text Word] OR "cameroun"[Text Word] OR "central african republic"[Text Word] OR "ubangi shari"[Text Word] OR "chad"[Text Word] OR "chile"[Text Word] OR "china"[Text Word] OR "colombia"[Text Word] OR "comoros"[Text Word] OR "comoro islands"[Text Word] OR "mayotte"[Text Word] OR "congo"[Text Word] OR "zaire"[Text Word] OR "costa rica"[Text Word] OR "cote d ivoire"[Text Word] OR "cote d ivoire"[Text Word] OR "cote d ivoire"[Text Word] OR "ivory coast"[Text Word] OR "croatia"[Text Word] OR "cuba"[Text Word] OR "cyprus"[Text Word] OR "czech republic"[Text Word] OR "czechoslovakia"[Text Word] OR "djibouti"[Text Word] OR "french somaliland"[Text Word] OR "dominica"[Text Word] OR "dominican republic"[Text Word] OR "ecuador"[Text Word] OR "egypt"[Text Word] OR "united arab republic"[Text Word] OR "el salvador"[Text Word] OR "equatorial guinea"[Text Word] OR "spanish guinea"[Text Word] OR "eritrea"[Text Word] OR "estonia"[Text Word] OR "eswatini"[Text Word] OR "swaziland"[Text Word] OR "ethiopia"[Text Word] OR "fiji"[Text Word] OR "gabon"[Text Word] OR "gabonese republic"[Text Word] OR "gambia"[Text Word] OR "georgia"[Text Word] OR "georgian"[Text Word] OR "ghana"[Text Word] OR "gold coast"[Text Word] OR "gibraltar"[Text Word] OR "greece"[Text Word] OR "grenada"[Text Word] OR "guam"[Text Word] OR "guatemala"[Text Word] OR "guinea"[Text Word] OR "guyana"[Text Word] OR "guiana"[Text Word] OR "haiti"[Text Word] OR "hispaniola"[Text Word] OR "honduras"[Text Word] OR "hungary"[Text Word] OR "india"[Text Word] OR "indonesia"[Text Word] OR "timor"[Text Word] OR "iran"[Text Word] OR "iraq"[Text Word] OR "isle of man"[Text Word] OR "jamaica"[Text Word] OR "jordan"[Text Word] OR "kazakhstan"[Text Word] OR "kazakh"[Text Word] OR "kenya"[Text Word] OR "korea"[Text Word] OR "kosovo"[Text Word] OR "kyrgyzstan"[Text Word] OR "kirghizia"[Text Word] OR "kirgizstan"[Text Word] OR "kyrgyz republic"[Text Word] OR "kirghiz"[Text Word] OR "laos"[Text Word] OR "lao pdr"[Text Word] OR "lao people s democratic republic"[Text Word] OR "latvia"[Text Word] OR "lebanon"[Text Word] OR "lesotho"[Text Word] OR "basutoland"[Text Word] OR "liberia"[Text Word] OR "libya"[Text Word] OR "libyan arab jamahiriya"[Text Word] OR "lithuania"[Text Word] OR "macau"[Text Word] OR "macao"[Text Word] OR "macedonia"[Text Word] OR "madagascar"[Text Word] OR "malagasy republic"[Text Word] OR "malawi"[Text Word] OR "nyasaland"[Text Word] OR "malaysia"[Text Word] OR "maldives"[Text Word] OR "indian ocean"[Text Word] OR "mali"[Text Word] OR "malta"[Text Word] OR "micronesia"[Text Word] OR "kiribati"[Text Word] OR "marshall islands"[Text Word] OR "nauru"[Text

Word] OR "northern mariana islands"[Text Word] OR "palau"[Text Word] OR "tuvalu"[Text Word] OR "mauritania"[Text Word] OR "mauritius"[Text Word] OR "mexico"[Text Word] OR "moldova"[Text Word] OR "moldovian"[Text Word] OR "mongolia"[Text Word] OR "montenegro"[Text Word] OR "morocco"[Text Word] OR "ifni"[Text Word] OR "mozambique"[Text Word] OR "portuguese east africa"[Text Word] OR "myanmar"[Text Word] OR "burma"[Text Word] OR "namibia"[Text Word] OR "nepal"[Text Word] OR "netherlands antilles"[Text Word] OR "nicaragua"[Text Word] OR "niger"[Text Word] OR "nigeria"[Text Word] OR "oman"[Text Word] OR "muscat"[Text Word] OR "pakistan"[Text Word] OR "panama"[Text Word] OR "papua new guinea"[Text Word] OR "paraguay"[Text Word] OR "peru"[Text Word] OR "philippines"[Text Word] OR "philipines"[Text Word] OR "phillipines"[Text Word] OR "phillippines"[Text Word] OR "poland"[Text Word] OR "polish people s republic"[Text Word] OR "portugal"[Text Word] OR "portuguese republic"[Text Word] OR "puerto rico"[Text Word] OR "romania"[Text Word] OR "russia"[Text Word] OR "russian federation"[Text Word] OR "ussr"[Text Word] OR "soviet union"[Text Word] OR "union of soviet socialist republics"[Text Word] OR "rwanda"[Text Word] OR "ruanda"[Text Word] OR "samoa"[Text Word] OR "pacific islands"[Text Word] OR "polynesia"[Text Word] OR "samoan islands"[Text Word] OR "sao tome and principe"[Text Word] OR "saudi arabia"[Text Word] OR "senegal"[Text Word] OR "serbia"[Text Word] OR "seychelles"[Text Word] OR "sierra leone"[Text Word] OR "slovakia"[Text Word] OR "slovak republic"[Text Word] OR "slovenia"[Text Word] OR "melanesia"[Text Word] OR "solomon island"[Text Word] OR "solomon islands"[Text Word] OR "norfolk island"[Text Word] OR "somalia"[Text Word] OR "south africa"[Text Word] OR "south sudan"[Text Word] OR "sri lanka"[Text Word] OR "ceylon"[Text Word] OR "saint kitts and nevis"[Text Word] OR "st kitts and nevis"[Text Word] OR "saint lucia"[Text Word] OR "st lucia"[Text Word] OR "saint vincent"[Text Word] OR "st vincent"[Text Word] OR "grenadines"[Text Word] OR "sudan"[Text Word] OR "suriname"[Text Word] OR "surinam"[Text Word] OR "syria"[Text Word] OR "syrian arab republic"[Text Word] OR "tajikistan"[Text Word] OR "tadjikistan"[Text Word] OR "tadzhikistan"[Text Word] OR "tadzhik"[Text Word] OR "tanzania"[Text Word] OR "tanganyika"[Text Word] OR "thailand"[Text Word] OR "siam"[Text Word] OR "timor leste"[Text Word] OR "east timor"[Text Word] OR "togo"[Text Word] OR "togolese republic"[Text Word] OR "tonga"[Text Word] OR "trinidad"[Text Word] OR "tobago"[Text Word] OR "tunisia"[Text Word] OR "turkey"[Text Word] OR "turkmenistan"[Text Word] OR "turkmen"[Text Word] OR "uganda"[Text Word] OR "ukraine"[Text Word] OR "uruguay"[Text Word] OR "uzbekistan"[Text Word] OR "uzbek"[Text Word] OR "vanuatu"[Text Word] OR "new hebrides"[Text Word] OR "venezuela"[Text Word] OR "vietnam"[Text Word] OR "viet nam"[Text Word] OR "middle east"[Text Word] OR "west bank"[Text Word] OR "gaza"[Text Word] OR "palestine"[Text Word] OR "yemen"[Text Word] OR "yugoslavia"[Text Word] OR "zambia"[Text Word] OR "zimbabwe"[Text Word] OR "northern rhodesia"[Text Word] OR "global south"[Text Word] OR "africa south of the sahara"[Text Word] OR "sub saharan africa"[Text Word] OR "subsaharan africa"[Text Word] OR "central africa"[Text Word] OR "north africa"[Text Word] OR "northern africa"[Text Word] OR "magreb"[Text Word] OR "maghrib"[Text Word] OR "sahara"[Text Word] OR "southern africa"[Text Word] OR "east africa"[Text Word] OR "eastern africa"[Text Word] OR "west africa"[Text Word] OR "western africa"[Text Word] OR "west indies"[Text Word] OR "indian ocean islands"[Text Word] OR "caribbean"[Text Word] OR "central america"[Text Word] OR "latin america"[Text Word] OR "south america"[Text Word] OR "central asia"[Text Word] OR "north asia"[Text Word] OR "northern asia"[Text Word] OR "southeastern asia"[Text Word] OR "south eastern asia"[Text Word] OR "southeast asia"[Text Word] OR "south east asia"[Text Word] OR "western asia"[Text Word]

OR "east europe"[Text Word] OR "eastern europe"[Text Word] OR "developing country"[Text Word] OR "developing countries"[Text Word] OR "developing nation"[Text Word] OR "developing nations"[Text Word] OR "developing population"[Text Word] OR "developing populations"[Text Word] OR "developing world"[Text Word] OR "less developed country"[Text Word] OR "less developed countries"[Text Word] OR "less developed nation"[Text Word] OR "less developed nations"[Text Word] OR "less developed world"[Text Word] OR "lesser developed countries"[Text Word] OR "lesser developed nations"[Text Word] OR "under developed country"[Text Word] OR "under developed countries"[Text Word] OR "under developed nations"[Text Word] OR "under developed world"[Text Word] OR "underdeveloped country"[Text Word] OR "underdeveloped countries"[Text Word] OR "underdeveloped nation"[Text Word] OR "underdeveloped nations"[Text Word] OR "underdeveloped population"[Text Word] OR "underdeveloped populations"[Text Word] OR "underdeveloped world"[Text Word] OR "middle income country"[Text Word] OR "middle income countries"[Text Word] OR "middle income nation"[Text Word] OR "middle income nations"[Text Word] OR "middle income population"[Text Word] OR "middle income populations"[Text Word] OR "low income country"[Text Word] OR "low income countries"[Text Word] OR "low income nation"[Text Word] OR "low income nations"[Text Word] OR "low income population"[Text Word] OR "low income populations"[Text Word] OR "lower income country"[Text Word] OR "lower income countries"[Text Word] OR "lower income nations"[Text Word] OR "lower income population"[Text Word] OR "lower income populations"[Text Word] OR "underserved countries"[Text Word] OR "underserved nations"[Text Word] OR "underserved population"[Text Word] OR "underserved populations"[Text Word] OR "under served population"[Text Word] OR "under served populations"[Text Word] OR "deprived countries"[Text Word] OR "deprived population"[Text Word] OR "deprived populations"[Text Word] OR "poor country"[Text Word] OR "poor countries"[Text Word] OR "poor nation"[Text Word] OR "poor nations"[Text Word] OR "poor population"[Text Word] OR "poor populations"[Text Word] OR "poor world"[Text Word] OR "poorer countries"[Text Word] OR "poorer nations"[Text Word] OR "poorer population"[Text Word] OR "poorer populations"[Text Word] OR "developing economy"[Text Word] OR "developing economies"[Text Word] OR "less developed economy"[Text Word] OR "less developed economies"[Text Word] OR "underdeveloped economies"[Text Word] OR "middle income economy"[Text Word] OR "middle income economies"[Text Word] OR "low income economy"[Text Word] OR "low income economies"[Text Word] OR "lower income economies"[Text Word] OR "low gdp"[Text Word] OR "low gnp"[Text Word] OR "low gross domestic"[Text Word] OR "low gross national"[Text Word] OR "lower gdp"[Text Word] OR "lower gross domestic"[Text Word] OR "lmic"[Text Word] OR "lmics"[Text Word] OR "third world"[Text Word] OR "lami country"[Text Word] OR "lami countries"[Text Word] OR "transitional country"[Text Word] OR "transitional countries"[Text Word] OR "emerging economies"[Text Word] OR "emerging nation"[Text Word] OR "emerging nations"[Text Word] OR "afghanistan"[MeSH Terms] OR "albania"[MeSH Terms] OR "algeria"[MeSH Terms] OR "american samoa"[MeSH Terms] OR "angola"[MeSH Terms] OR "antigua and barbuda"[MeSH Terms] OR "argentina"[MeSH Terms] OR "armenia"[MeSH Terms] OR "aruba"[MeSH Terms] OR "azerbaijan"[MeSH Terms] OR "bahrain"[MeSH Terms] OR "bangladesh"[MeSH Terms] OR "barbados"[MeSH Terms] OR "republic of belarus"[MeSH Terms] OR "belize"[MeSH Terms] OR "benin"[MeSH Terms] OR "bhutan"[MeSH Terms] OR "bolivia"[MeSH Terms] OR "bosnia and herzegovina"[MeSH Terms] OR "botswana"[MeSH Terms] OR "brazil"[MeSH Terms] OR "bulgaria"[MeSH Terms] OR "burkina faso"[MeSH Terms] OR "burundi"[MeSH Terms] OR "cabo verde"[MeSH Terms] OR "cambodia"[MeSH Terms] OR "cameroon"[MeSH Terms] OR "central african

republic"[MeSH Terms] OR "chad"[MeSH Terms] OR "chile"[MeSH Terms] OR "china"[MeSH Terms] OR "colombia"[MeSH Terms] OR "comoros"[MeSH Terms] OR "democratic republic of the congo"[MeSH Terms] OR "congo"[MeSH Terms] OR "costa rica"[MeSH Terms] OR "cote d ivoire"[MeSH Terms] OR "croatia"[MeSH Terms] OR "cuba"[MeSH Terms] OR "cyprus"[MeSH Terms] OR "czech republic"[MeSH Terms] OR "djibouti"[MeSH Terms] OR "dominica"[MeSH Terms] OR "dominican republic"[MeSH Terms] OR "ecuador"[MeSH Terms] OR "egypt"[MeSH Terms] OR "el salvador"[MeSH Terms] OR "equatorial guinea"[MeSH Terms] OR "eritrea"[MeSH Terms] OR "estonia"[MeSH Terms] OR "eswatini"[MeSH Terms] OR "ethiopia"[MeSH Terms] OR "fiji"[MeSH Terms] OR "gabon"[MeSH Terms] OR "gambia"[MeSH Terms] OR "georgia republic"[MeSH Terms] OR "ghana"[MeSH Terms] OR "gibraltar"[MeSH Terms] OR "greece"[MeSH Terms] OR "grenada"[MeSH Terms] OR "guam"[MeSH Terms] OR "guatemala"[MeSH Terms] OR "guinea"[MeSH Terms] OR "guinea bissau"[MeSH Terms] OR "guyana"[MeSH Terms] OR "haiti"[MeSH Terms] OR "honduras"[MeSH Terms] OR "hungary"[MeSH Terms] OR "india"[MeSH Terms] OR "indonesia"[MeSH Terms] OR "iran"[MeSH Terms] OR "iraq"[MeSH Terms] OR "jamaica"[MeSH Terms] OR "jordan"[MeSH Terms] OR "kazakhstan"[MeSH Terms] OR "kenya"[MeSH Terms] OR "democratic people s republic of korea"[MeSH Terms] OR "republic of korea"[MeSH Terms] OR "kosovo"[MeSH Terms] OR "kyrgyzstan"[MeSH Terms] OR "laos"[MeSH Terms] OR "latvia"[MeSH Terms] OR "lebanon"[MeSH Terms] OR "lesotho"[MeSH Terms] OR "liberia"[MeSH Terms] OR "libya"[MeSH Terms] OR "lithuania"[MeSH Terms] OR "macau"[MeSH Terms] OR "republic of north macedonia"[MeSH Terms] OR "madagascar"[MeSH Terms] OR "malawi"[MeSH Terms] OR "malaysia"[MeSH Terms] OR "indian ocean islands"[MeSH Terms] OR "mali"[MeSH Terms] OR "malta"[MeSH Terms] OR "micronesia"[MeSH Terms] OR "palau"[MeSH Terms] OR "mauritania"[MeSH Terms] OR "mauritius"[MeSH Terms] OR "mexico"[MeSH Terms] OR "moldova"[MeSH Terms] OR "mongolia"[MeSH Terms] OR "montenegro"[MeSH Terms] OR "morocco"[MeSH Terms] OR "mozambique"[MeSH Terms] OR "myanmar"[MeSH Terms] OR "namibia"[MeSH Terms] OR "nepal"[MeSH Terms] OR "netherlands antilles"[MeSH Terms] OR "nicaragua"[MeSH Terms] OR "niger"[MeSH Terms] OR "nigeria"[MeSH Terms] OR "oman"[MeSH Terms] OR "pakistan"[MeSH Terms] OR "panama"[MeSH Terms] OR "papua new guinea"[MeSH Terms] OR "paraguay"[MeSH Terms] OR "peru"[MeSH Terms] OR "philippines"[MeSH Terms] OR "poland"[MeSH Terms] OR "portugal"[MeSH Terms] OR "puerto rico"[MeSH Terms] OR "romania"[MeSH Terms] OR "russia"[MeSH Terms] OR "rwanda"[MeSH Terms] OR "samoa"[MeSH Terms] OR "sao tome and principe"[MeSH Terms] OR "saudi arabia"[MeSH Terms] OR "senegal"[MeSH Terms] OR "serbia"[MeSH Terms] OR "seychelles"[MeSH Terms] OR "sierra leone"[MeSH Terms] OR "slovakia"[MeSH Terms] OR "slovenia"[MeSH Terms] OR "melanesia"[MeSH Terms] OR "somalia"[MeSH Terms] OR "south africa"[MeSH Terms] OR "south sudan"[MeSH Terms] OR "sri lanka"[MeSH Terms] OR "saint kitts and nevis"[MeSH Terms] OR "saint lucia"[MeSH Terms] OR "saint vincent and the grenadines"[MeSH Terms] OR "sudan"[MeSH Terms] OR "suriname"[MeSH Terms] OR "syria"[MeSH Terms] OR "tajikistan"[MeSH Terms] OR "tanzania"[MeSH Terms] OR "thailand"[MeSH Terms] OR "timor leste"[MeSH Terms] OR "togo"[MeSH Terms] OR "tonga"[MeSH Terms] OR "trinidad and tobago"[MeSH Terms] OR "tunisia"[MeSH Terms] OR "turkey"[MeSH Terms] OR "turkmenistan"[MeSH Terms] OR "uganda"[MeSH Terms] OR "ukraine"[MeSH Terms] OR "uruguay"[MeSH Terms] OR "uzbekistan"[MeSH Terms] OR "vanuatu"[MeSH Terms] OR "venezuela"[MeSH Terms] OR "vietnam"[MeSH Terms] OR "middle east"[MeSH Terms] OR "yemen"[MeSH Terms] OR "yugoslavia"[MeSH Terms] OR "zambia"[MeSH Terms] OR "zimbabwe"[MeSH Terms] OR "africa south of the sahara"[MeSH Terms] OR "africa, central"[MeSH Terms] OR "africa,

northern"[MeSH Terms] OR "africa, southern"[MeSH Terms] OR "africa, eastern"[MeSH Terms] OR "africa, western"[MeSH Terms] OR "west indies"[MeSH Terms] OR "indian ocean islands"[MeSH Terms] OR "caribbean region"[MeSH Terms] OR "central america"[MeSH Terms] OR "latin america"[MeSH Terms] OR "south america"[MeSH Terms] OR "asia, central"[MeSH Terms] OR "asia, northern"[MeSH Terms] OR "asia, southeastern"[MeSH Terms] OR "asia, western"[MeSH Terms] OR "europe, eastern"[MeSH Terms] OR "developing countries"[MeSH Terms])

Category 2: RMNCH

"Reproductive Health Services"[Mesh] OR "reproductive health service*"[tw] or "family planning"[tw] or "planned pregnanc*"[tw] OR "Women's Health"[Mesh] OR "Maternal-Child Health Services"[Mesh] OR "Maternal-Child Health Centers"[Mesh] OR "Maternal Child Health Service*"[tw] OR "Maternal Child Health Center*"[tw] OR "womens health"[tw] OR "contraception" OR "Contraception"[Mesh] OR "Fertil*"[tw] OR "Birth Control"[tw] or "Contracept*"[tw] OR "Infant Health"[Mesh]

Category 3: Providers

"Community Health Workers"[Mesh] OR "Health Personnel"[Mesh] OR Health Care Provider*[tw] OR Healthcare Provider*[tw] OR Healthcare Worker* [tw] OR health care worker*[tw] OR healthcare professional*[tw] OR health care professional*[tw] OR ((Accredited Social Health Activist) OR (ASHA)) OR (Community Health Extension Worker)

Category 4: Provider behavioral and behavioral determinants

"Behavior and Behavior Mechanisms"[Mesh] OR "behavior and behavior mechanism*"[tw] OR "health personnel attitude*"[tw] OR "staff attitude*"[tw] OR "motivation*"[tw] OR "disincentive*"[tw] OR "Expectation*"[tw] OR "Incentive*"[tw] OR "Acceptance Process*"[tw] OR "counseling"[tw] OR "Behavior*"[tw] OR "social determinant*"[tw] OR "structural determinant*"[tw] OR ("Attitude of Health Personnel"[Mesh] OR "health personnel attitude*"[tw] OR "staff attitude*"[tw]) OR "Employee Performance Appraisal"[Mesh] OR "Quality of Health Care"[Mesh] OR "Professional-Patient Relations"[Mesh] OR "Diagnosis"[Mesh] OR "Outcome and Process Assessment, Health Care"[Mesh] OR "Therapeutics"[Mesh] OR "Referral and Consultation"[Mesh] OR "Attentional Bias"[Mesh] OR "Prejudice"[Mesh] OR "Social Support"[Mesh]

Category 5: Provider behavior change interventions

("Organization and Administration"[Mesh] OR "Personnel Management"[Mesh] OR "Staff Development"[Mesh] OR "Inservice Training"[Mesh] OR "Education"[Mesh] OR Values clarification OR "Facility Regulation and Control"[Mesh] OR "Health Policy"[Mesh] OR "Organizational Innovation"[Mesh] OR "Quality Improvement"[Mesh] OR "Motivation"[Mesh] OR infrastructure improvement OR "Environment Design"[Mesh] OR "Social Responsibility"[Mesh] AND (humans[Filter])) AND (humans[Filter])) AND (humans[Filter]))
